# Precision Lasso: accounting for correlations and linear dependencies in high-dimensional genomic data

**DOI:** 10.1093/bioinformatics/bty750

**Published:** 2018-09-01

**Authors:** Haohan Wang, Benjamin J Lengerich, Bryon Aragam, Eric P Xing

**Affiliations:** 1 Language Technologies Institute, School of Computer Science, Carnegie Mellon University, Pittsburgh, PA, USA; 2 Department of Computer Science, School of Computer Science, Carnegie Mellon University, Pittsburgh, PA, USA; 3 Department of Machine Learning, School of Computer Science, Carnegie Mellon University, Pittsburgh, PA, USA

## Abstract

**Motivation:**

Association studies to discover links between genetic markers and phenotypes are central to bioinformatics. Methods of regularized regression, such as variants of the Lasso, are popular for this task. Despite the good predictive performance of these methods in the average case, they suffer from *unstable* selections of correlated variables and *inconsistent* selections of linearly dependent variables. Unfortunately, as we demonstrate empirically, such problematic situations of correlated and linearly dependent variables often exist in genomic datasets and lead to under-performance of classical methods of variable selection.

**Results:**

To address these challenges, we propose the Precision Lasso. Precision Lasso is a Lasso variant that promotes sparse variable selection by regularization governed by the covariance and inverse covariance matrices of explanatory variables. We illustrate its capacity for *stable* and *consistent* variable selection in simulated data with highly correlated and linearly dependent variables. We then demonstrate the effectiveness of the Precision Lasso to select meaningful variables from transcriptomic profiles of breast cancer patients. Our results indicate that in settings with correlated and linearly dependent variables, the Precision Lasso outperforms popular methods of variable selection such as the Lasso, the Elastic Net and Minimax Concave Penalty (MCP) regression.

**Availability and implementation:**

Software is available at https://github.com/HaohanWang/thePrecisionLasso.

**Supplementary information:**

Supplementary data are available at *Bioinformatics* online.

## 1 Introduction

High-throughput technology for profiling gene expression levels and assaying genetic variations at a genome-wide scale produces massive data, creating an opportunity to study the genetic causes of complex diseases by statistical methods. Computationally screening for putatively causal genes is a key step in hypothesis generation, and many such techniques have been proposed. These can be categorized into several generations, starting with hypothesis testing (Posada and Crandall, 1998). Unfortunately, traditional hypothesis testing methods are limited to independently considering associations for each biomarker. This is a major limitation of the approach since epistatic effects remove the assumed independence between explanatory variables.

To jointly consider associations between all biomarkers and a phenotype, linear regression-based methods (Ogutu *et al.*, 2012) have become more popular. Although ordinary least squares regression can consider multiple genes simultaneously, it assigns non-zero effect sizes to all explanatory variables and fails when there are more genes than samples under consideration, the *high-dimensional* regime that is common in genomic applications. To solve this problem, regularization via the Lasso (Tibshirani, 1996) is often used to reduce the selected set of explanatory variables. Given a design matrix *X* (of size *n *×* p*, with $X_{i}^{j}$ the *j*th variable of the *i*th sample) and dependent variable *Y* (of size *n *×* *1), the Lasso solves the problem

$$\underset{\beta }{\arg\;\min}\frac{1}{2}||Y-X\beta |{|}_{2}^{2}\;\;\text{subject}\;\text{to}\;||\beta |{|}_{1}\leq t$$

where β represents the effect sizes of the explanatory variables and *t *>* *0 controls the amount of regularization. With the $\ell_{1}$-norm as a constraint, the Lasso learns a set of sparse coefficients β that indicates the most relevant explanatory variables. However, the Lasso has several drawbacks for structured data; here, we describe two situations that lead to undesirable properties in sparse variable selection: correlation and linear dependence between explanatory variables.

First, if two explanatory variables are highly correlated and effect sizes are unconstrained, then the explanatory variables show very similar influence on the response variable. In such a situation, the Lasso will only select one variable at random (Xu *et al.*, 2012). This is problematic when the results are used for hypothesis generation because we would like to simultaneously select all variables which have the same evidence of activity. Here, we refer to this property as *instability*.

Second, the Lasso struggles when explanatory variables are linearly dependent. Given explanatory variables *X^i^*, *X^j^*, *X^k^* with effect sizes β_*i*_, β_*j*_, β_*k*_, if $X^{k}=aX^{i}+cX^{j}$ and *a*β_*i*_ ≥ 0 and *c*β_*j*_ ≥ 0, then the Lasso is guaranteed to select the combined variable *X^k^* when *ac* > 0 (Zhao and Yu, 2006). This is undesirable when *X_i_*, *X_j_* are better experimental targets; for instance, *X_i_* and *X_j_* may be somatic mutations and *X_k_* a protein expression level. Here, we refer to this property as the *inconsistency* of the Lasso, following the convention of previous work (Zhao and Yu, 2006). Although linear dependence may involve arbitrarily many variables (and this is indeed an issue in practice), in the special case of two variables, linearly dependent variables may also be understood as the limiting case of perfect correlation.

Until now, these two properties have not been addressed simultaneously in a satisfactory manner. Unfortunately, as we will show, these two properties are common in genomic datasets and degrade performance in inference tasks such as the detection of putatively causal mutations. In this paper, we aim to address these two properties by introducing a new variable selection method called the Precision Lasso. We demonstrate empirically that our proposed model can mitigate the *instability* and *inconsistency* properties discussed here.

The main contributions of this paper are 3-fold:

- We demonstrate that real-world genomic datasets contain highly correlated and linearly dependent variables, raising concerns about *instability* and *inconsistency* for existing variable selection methods.
- We illustrate through experiments that these two properties degrade the performance of traditional variable selection methods.
- We propose a novel penalization to handle these properties and show that it outperforms traditional methods on simulated data and real breast cancer transcriptomic data.

### 1.1 Related work

It is well-known that the $\ell_{0}$-norm regularizer is optimal in variable selection, however, it leads to a non-convex programme which is NP-hard (Barron *et al.*, 1999; Davis *et al.*, 1997; Manyem and Ugon, 2012). To overcome this difficulty, $\ell_{1}$-norm regularization was proposed by Tibshirani (1996) as a tractable convex relaxation to $\ell_{0}$-regularization. Despite its many attractive properties (e.g. good predictive power), $\ell_{1}$-regularization—or *Lasso* regression—still suffers from the unstable and inconsistency properties mentioned in the previous section. The adaptive Lasso (Zou, 2006) aims to remedy some of the issues with vanilla $\ell_{1}$–regularization. This method re-weights the Lasso penalty for each variable based on the variable’s contribution in unregularized linear regression, and leads to more favourable variable selection properties. Unfortunately, the adaptive Lasso has also been shown to perform poorly in the presence of highly correlated variables (Krämer *et al.*, 2009).

Another popular alternative is $\ell_{2}$-norm regularization, often called ridge regression or Tikhonov regularization (Golub *et al.*, 1999; Hoerl and Kennard, 1970), however, this strategy loses the attractive variable selection properties of the Lasso. There are also some works that aim to combine the advantages of $\ell_{1}$- and $\ell_{2}$-regularization, such as the elastic net (Zou and Hastie, 2005) and the trace Lasso (Grave *et al.*, 2011). These two approaches are designed to handle correlated variables, but they have no guarantees for linearly dependent variables. In other words, these methods can select variables *stably*, but not *consistently*. We also mention the non-negative Garrote (Yuan and Lin, 2007); however, this is only applicable when *p *<* n* and therefore is not applicable to most tasks in bioinformatics.

Another approach to overcome these difficulties is to use non-convex regularizers, introduced by Fan and Li (2001). Examples include the Smoothly Clipped Absolute Deviation (SCAD) (Fan and Li, 2001) and the Minimax Concave Penalty (MCP) (Zhang, 2010). These non-convex regularizers are designed to overcome the problems inherent with the Lasso, and have the desirable properties of unbiasedness, continuity and sparsity. A recent review of these methods can be found in (Zhang and Zhang, 2012). While these variable selection methods are promising compared to the Lasso, we will show that they also inherit many of the Lasso’s problems in practice.

In addition to these general purpose algorithms, additional methods tailored specifically for GWAS have been developed. To tackle the high-dimensionality of genomic datasets, Wu *et al.* (2009) reduced the dimension of SNPs via a simple score criterion, then applied the LASSO to the reduced set. He and Lin (2011) extended this approach based on Fan and Lv (2008) with a more sophisticated score criterion where the score is conditioned on SNPs that were selected previously. Bayesian variable selection methods (Guan and Stephens, 2011; Peltola *et al.*, 2012) have also enjoyed recent popularity for selecting SNPs in GWAS. Unfortunately, none of these methods explicitly address the problems raised by *inconsistent* selection.

### 1.2 Motivation

Gene expression profiles can result in unstable variable selection. As expression levels of genes within a regulatory pathway are highly correlated (Michalopoulos *et al.*, 2012), the Lasso will be *unstable* for selection of variables when several of the variables participate in the same regulatory pathway. This can lead researchers to believe that a single element of the pathway is likely to be causal for a phenotype (based on variable selection), when in reality the evidence is shared between all elements of the pathway.

The existence of expression profiles that lead to *inconsistent* behaviour, however, has not been convincingly demonstrated previously. Here, we verify that real gene expression profiles indeed exhibit linear dependencies, thereby introduce the problem of *inconsistency*.

To show this, we must first formalize the definition of *inconsistency*. We call a explanatory variable an *active variable* if the explanatory variable encodes an interaction that influences the value of the response variable. In a linear regression model for these variables, this means that the coefficient for this variable is non-zero. For the Lasso to select *consistently*, data must satisfy the *irrepresentable condition* (Ravikumar *et al.*, 2010; Zhao and Yu, 2006), which states that the association between the active variables and the non-active variables cannot be too strong. Formally, the *irrepresentable condition* states that

(1) $$|{\left(X^{\left(2\right)}\right)}^{T}X^{\left(1\right)}{\left({\left(X^{\left(1\right)}\right)}^{T}X^{\left(1\right)}\right)}^{-1}\text{sign}(\beta^{\left(1\right)})|<1-\eta ,$$

where *X*^(1)^ is the set of active variables, *X*^(2)^ is the set of non-active variables, **1** is a vector of ones, η is a positive constant vector, $\text{sign}(\beta^{\left(1\right)})$ stands for the sign of the coefficients of active variables, and the inequality holds element-wise.

A matrix which breaks this condition called *non-irrepresentable* (Zhao and Yu, 2006), and leads to *inconsistent* variable selection. In order to undertand how serious of an issue this is in practice, we studied the non-irrepresentable condition across three types of real genomic data for three cancer types. All data comes from TCGA (http://cancergenome.nih.gov/). These datasets are moderately large: 617, 504 and 595 patients for the Glioblastoma, Lung and Breast datasets, respectively. The datasets also contain a high number of explanatory variables: 17 814 for gene expression, 27 577 for methylation and 4201 for miRNA. As the data are drawn from multiple assay and cancer types, they can be considered a representative sample of genomic datasets one might encounter in practice.

**Fig. 1.**
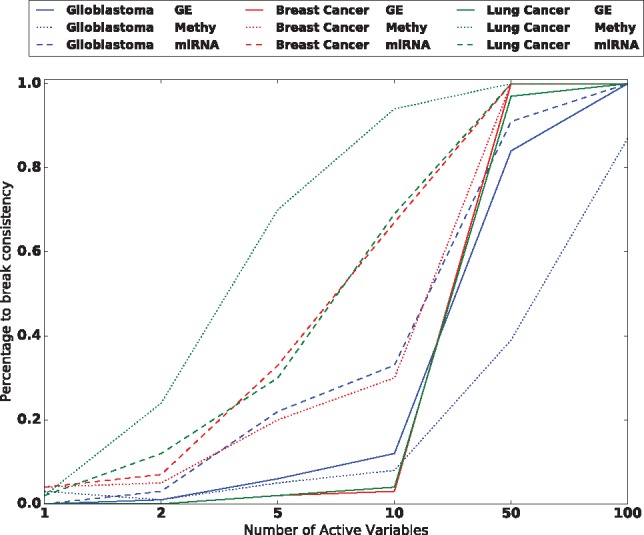
Proportion of simulations in which the *irrepresentable* condition failed to hold on gene expression, methylation, miRNA datasets for glioblastoma, breast cancer and lung cancer

We tested the non-irrepresentable condition on these datasets as follows: Using the real data as the design matrix *X*, we selected *K* random genes to be ‘active’ in a simulated linear regression model. Then we checked the irrepresentability condition Equation (1) with η* *= 10^– 5^ based on this active set. We replicated this 100 times for K=1,2,5,10,50,100 and Figure 1 illustrates the proportion of simulations that were non-irrepresentable and hence *inconsistent* for the Lasso. In all datasets, at least one active set broke this condition for each *K* and for *K *>* *10, almost all active sets broke this condition.

To check the *instability* condition, we also calculated the frequency of highly correlated variables in the data. Six of the nine datasets checked had at least one pair of variables that had an empirical correlation coefficient of 0.99 or higher. These experiments indicate that both non-irrepresentability and high correlation—and hence *inconsistency* and *instability*—are both prevalent and a serious nuisance in working with real genomic datasets, motivating the need for more powerful methods that are robust to these problems.

## 2 Materials and methods

In this section, we introduce the Precision Lasso, a regularized regression method that mitigates the problems exposed in the previous section. We first introduce techniques to handle *instability* and *inconsistency* separately, and then combine these two approaches in order to derive the final model.

### 2.1 *Instability*: dealing with correlated variables

As introduced previously, the *instability* of the Lasso refers to its inability to distinguish the effects of correlated explanatory variables. Since correlated explanatory variables cannot be separated by observational statistics, one solution is to force the model to simultaneously identify correlated variables. A straightforward way to fulfil this goal is to assign similar weights to correlated variables.

A convenient set of weights to use are the sum of each explanatory variable’s correlation with other explanatory variables. Following the trace Lasso (Grave *et al.*, 2011), this can be achieved by solving

(2) $$\underset{\beta }{\arg\;\min}\frac{1}{2}||Y-X\beta |{|}_{2}^{2}+\lambda ||{\left(X^{T}X\right)}^{\frac{1}{2}}\text{diag}(\beta )|{|}_{*},$$

where $||\;\cdot \;|{|}_{*}$ denotes the trace norm, which is defined as the sum of the singular values of a matrix (Srebro and Shraibman, 2005). This regularizer accounts for the correlation between variables and ensures sparsity in the final model while maintaining stable selection.

### 2.2 *Inconsistency*: dealing with linear dependencies

Next we extend this regularizer to consider *inconsistency*. First, observe that Equation (1) can be rewritten as follows:

$$\phi^{\left(1\right)}<1-\eta ,\;\text{where}\;X^{\left(2\right)}=X^{\left(1\right)}\phi^{\left(1\right)}.$$

Evidently the *irrepresentable condition* breaks when $\phi^{\left(1\right)}\geq 1-\eta$, i.e. when there is a linear relationship between *X*^(1)^ and *X*^(2)^ with sufficiently large coefficients.

Following a similar strategy as in Section 2.1, we re-weight the regularizers with the sum of each explanatory variable’s linear regression coefficients towards the other explanatory variables. Since these coefficients are unknown, we propose to use the inverse covariance matrix as a surrogate, owing to the well-known relationship between partial regression coefficients and the inverse covariance matrix (Cramer, 1946; Dempster, 1969). Recall that the partial regression coefficients express the linear dependence between a variable *X^i^* and the rest of the variables. Thus, in order to address *inconsistency*, we use the inverse covariance structure in an analogous manner to Equation (2), resulting in the following regularized model:

(3) $$\underset{\beta }{\arg\;\min}\frac{1}{2}||Y-X\beta |{|}_{2}^{2}+\lambda ||{\left(X^{T}X+\mu I\right)}^{-\frac{1}{2}}\text{diag}(\beta )|{|}_{*}.$$

Here, μ is a small positive parameter that is used to make the singular matrix *X^TX^* invertible when *p *>* n*. In our experiments, we follow the protocol of Grave *et al.* to select μ (details in Supplementary Material).

### 2.3 Precision lasso

To simultaneously consider both *inconsistency* and *instability*, we propose to combine the two regularization schemes proposed in the previous sections. To this end, we employ an additional hyperparameter γ in order to tune the regularizer to pay more attention to *instability* (i.e. the *X^TX^* term) or to *inconsistency* (i.e. the ${\left(X^{T}X+\mu I\right)}^{-\frac{1}{2}}$ term):

(4) $$\begin{array}{c} \underset{\beta }{\arg\;\min}\frac{1}{2}||Y-X\beta |{|}_{2}^{2}\\ +\lambda ||[\gamma {\left(X^{T}\;X\right)}^{\frac{1}{2}}+(1-\gamma ){\left(X^{T}\;X+\mu I\right)}^{-\frac{1}{2}}]\text{diag}(\beta )|{|}_{*}. \end{array}$$

The choice of γ* *= 1∕2 gives equal weight to each problem. As the inverse covariance matrix is also known as the precision matrix, we name the proposed model in Equation (4) the *Precision Lasso*.

### 2.4 Precision lasso for binary response

The regularization strategy introduced in the previous section can be extended to other convex cost functions ℓ in a straightforward manner:

$$\begin{array}{c} \underset{\beta }{\arg\;\min}\;\ell (X,y;\beta )\\ +\lambda ||[\gamma {\left(X^{T}\;X\right)}^{\frac{1}{2}}+(1-\gamma ){\left(X^{T}\;X+\mu I\right)}^{-\frac{1}{2}}]\text{diag}(\beta )|{|}_{*} \end{array}$$

For example, when the response of the data is binary, the Precision Lasso can be applied to the logistic cost function by replacing ℓ with the negative log-likelihood of the logistic regression model. This formulation will be exploited in our experiment on case-control data. Furthermore, similar irrepresentability-type conditions are necessary for consistency in this more general setting (e.g. Ravikumar *et al.*, 2010).

### 2.5 Learning algorithm

In order to solve for β in Equation (4), we first derive an upper bound on the Precision Lasso cost function which will be used as a computationally efficient surrogate of the original formulation. The details of this derivation can be found in the Supplementary Material. Thus, instead of optimizing Equation (4) directly, we optimize the resulting surrogate:

$$\begin{array}{c} \underset{\beta }{\arg\;\min}||y-X\beta |{|}_{2}^{2}\\ +\gamma ||{\left[(X\text{diag}(\beta ))T(X\text{diag}(\beta ))\right]}^{\frac{1}{2}}|{|}_{*}\\ +(1-\gamma )||[(X\text{diag}(\beta^{-1}))T(X\text{diag}(\beta^{-1}))+\mu I]-\frac{1}{2}|{|}_{*} \end{array}$$

Finally, to solve this optimization problem, we employ an iteratively re-weighted least squares algorithm, which is a standard algorithm for solving problems of this form; for details see Supplementary Section S2 of the Supplementary Material.

## 3 Results

In this section, we validate the performance of our proposed Precision Lasso algorithm by comparing it to other variable selection methods. We compare the full Precision Lasso (PL) to a lightweight version that only uses the inverse covariance matrix in the regularizer (IC), which amounts to setting γ* *= 0. This version effectively considers only linear dependent variables, and helps to contrast the added benefits of considering *both* properties versus either alone. We also compare to the following baselines: Wald Hypothesis Testing, Sure Independence Screening (SIS) (Fan and Lv, 2008), Lasso regression, Ridge regression (RR), Elastic Net (EN), Adaptive Lasso (AL), SCAD, MCP and trace Lasso (TL). For the adaptive Lasso, we used the method introduced in Huang *et al.* (2008) to allow it to be applied to high-dimensional data. As the non-negative garotte does not work in the high dimension regime (Yuan and Lin, 2007), it was not included in our simulations.

To compare these methods, we ran the following experiments: (i) Simulated data with a continuous response, (ii) simulated case-control data using logistic regression and (3) breast cancer gene expression data. We report here the results for binary case-control data, although the results for continuous data are similar (details of all the experiments can be found in the Supplementary Material).

### 3.1 Simulation data

To simulate input data with high correlation between covariates, we use an auto-regressive sampling scheme (see Supplementary Sections S3.1 for details). We report here the average AUC score (area under ROC curve) for case-control data. Following the convention of Wu *et al.* (2009) to select the parameter λ (the weight of regularizer) by the number of selected variables, we select exactly *K *=* k* variables where *k* is the number of active variables in synthetic data. This helps to avoid overly complex models, which tend to selected by automatic measures such as cross-validation and AIC/BIC (Meinshausen and Bühlmann, 2006). Moreover, it is well-known that prediction performance and parameter estimation performance are not directly related. For example, the *unstable* and *inconsistent* problems that are the main focus of this paper illustrates this point: Good performance in prediction may be achieved by selecting correlated variables or linearly dependent variables, even though these variables may not be directly related to the response. For the Precision Lasso, the extra parameter γ is set as the prevalence of correlated variables divided by the prevalence of linearly dependent variables.

**Fig. 2.**
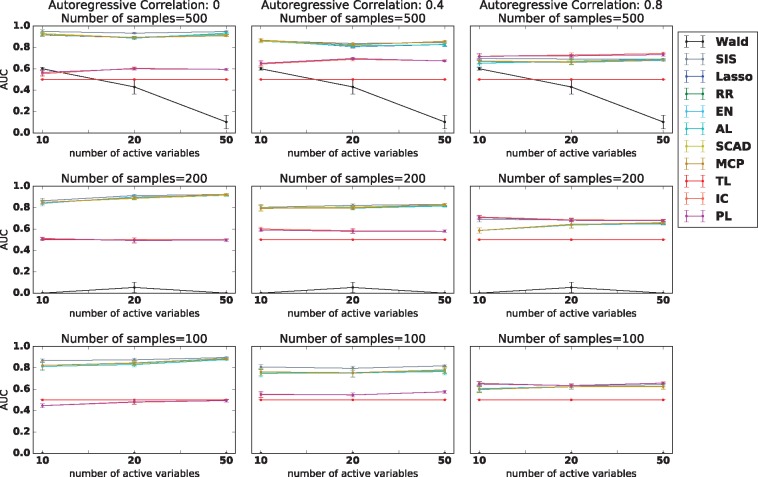
AUC of each variable selection method. Methods are: Wald Hypothesis Testing (Wald), Sure Independence Screening (SIS), Lasso, Ridge Regression (RR), Elastic Net (EN), Adaptive Lasso (AL), SCAD, MCP, Trace Lasso (TL), Inverse Covariance Regularizer (IC) and Precision Lasso (PL). The vertical axis represents area under ROC of the variable selection. The results are averaged from ten runs and SD is also shown. From the plot, we can see that our methods (PL and IC) exhibit a clear advantage over traditional methods on simulation data. Please notice that the AUC is calculated for variable selection task, instead of prediction of binary outcomes

On data simulated from a binary logistic model, we see higher AUC for the Precision Lasso methods than for the baseline methods in the cases when auto-regressive coefficient is strong (Fig. 2). Interestingly, we can see that in the case where auto-regressive correlation is low (i.e. the setting for which the Precision Lasso was not originally intended), the Precision Lasso performs inferior to other methods. We further test for many other evaluation criterion including true positives, false positives, precision, recall and F1 score. The entire table can be found in the Supplementary Material (Supplementary Table S1). The Precision Lasso shows superior performance over the competing methods when the auto-regressive correlation is high.

We also tested the performance of these methods for continuous response data. Here, we observed that the Precision Lasso outperformed other methods, even more so relative to our experiments on case-control data (Supplementary Fig. S2). Similar to case-control data, thePrecision Lasso behaves slightly worse than other competing methods when the correlation is low, but achieves clearly better performance than competing methods when correlation is high. Additionally, one may notice that SIS behaves only slightly worse than Precision Lasso in Figure 2 on case-control data, but in Supplementary Figure S2, we observe a clear advantage of Precision Lasso over SIS. The gap between these two methods can be more clearly compared in Supplementary Table S2, where we present a detailed evaluation of all the methods along with other metrics.

We also tested Precision Lasso in several other settings, including mis-specified number of active variables (i.e. *K *=* k*∕2 and *K *=* *2*k*). We see that the Precision Lasso again outperforms the baseline methods in settings of large auto-correlation. Finally, we also compare the strategies for tuning hyperparameters to verify the argument that cross-validation is a less favourable parameter tuning strategy for variable selection. Detailed results are reported in Supplementary Section S6 of the Supplementary Material.

### 3.2 Breast cancer transcriptomic data

#### 3.2.1 Data

To investigate the performance of Precision Lasso on real genomic data, we use breast cancer mRNA expression data from TCGA (http://cancergenome.nih.gov/) as explanatory variables and the case-control status as the phenotype. These data consist of RNA-seq assays for 532 breast cancer samples and 63 matching control samples. While it is typical to perform significant pre-processing on these data, here we are interested in the performance of statistical methods to extract signal from data with structured noise. For this reason, we perform variable selection on the FPKM-normalized RNA-seq counts of 10 000 genes.

#### 3.2.2 Evaluation

For each variable selection method, we select exactly 100 genes. To evaluate the quality of these selections, we seek to identify genes that are likely to be involved in the causation of the breast cancer. First, we filter the selected genes by intersection with the Catalogue of Somatic Mutations in Cancer (Forbes *et al.*, 2015) (COSMIC). Next, we use the results of a recent analysis (Rajendran and Deng, 2017) which combined annotations in COSMIC, IntOGen (Gonzalez-Perez *et al.*, 2013), CBioPortal (Cerami *et al.*, 2012) and OASIS (http://oasis-genomics.org/) to generate a list of potential driver mutations in breast cancer and cross-check each selected gene for evidence of driver function. The results are shown in Table 1.

**Table 1.** Genes that were selected from breast cancer gene expression data and are annotated in the COSMIC dataset to have somatic mutations associated with tumours

Method	Selected gene	Tumor associations	Driver?
Precision lasso	* ****FOXA1***	**Breast, Prostate**	*✓*
	* AR*	Prostate	*✓*
	* PBX1*	pre B-cell ALL, Myoepithelioma	
	* COX6C*	Uterine leiomyoma	
Wald test	* PPARG*	Follicular thyroid	
	* EBF1*	Lipoma	
	*** TPM3***	**Papillary thyroid; ALCL; NSCLC; Spitzoid tumour**	
Lasso	* HMGA2*	Lipoma; Leiomyoma; Pleomorphic salivary gland adenoma	
	* COL1A1*	DFSP, Aneurysmal bone cyst	
Ridge regression	* PPARG*	Follicular thyroid	
	* COL1A1*	DFSP, Aneurysmal bone cyst	
Elastic net	* HMGA2*	Lipoma; Leiomyoma; Pleomorphic salivary gland adenoma	
	* COL1A1*	DFSP, Aneurysmal bone cyst	
Adaptive lasso	* CBLC*	Acute Myeloid leukaemia	
	* HMGA2*	Lipoma; Leiomyoma; Pleomorphic salivary gland adenoma	
	* COL1A1*	DFSP, Aneurysmal bone cyst	
SCAD	* GATA1*	Megakaryoblastic leukaemia of downs syndrome	
	* FCGR2B*	Acute lymphoblastic leukaemia	
	* HMGA2*	Lipoma; Leiomyoma; Pleomorphic salivary gland adenoma	
MCP	* MYH11*	Acute myeloid leukaemia	
	* HMGA2*	Lipoma; Leiomyoma; Pleomorphic salivary gland adenoma	
	* COL1A1*	DFSP, Aneurysmal bone cyst	
Trace lasso	*** FOXA1***	**Breast, Prostate**	*✓*
	* EBF1*	Lipoma	
	* CDKN2A*	Melanoma	
	* COL1A1*	DFSP, Aneurysmal bone cyst	
Inverse covariance	*** RAC1***	**Carcinoma, Melanoma**	
	* TPR*	Papillary thyroid, NSCLC	
	* ZNF384*	Acute lymphoblastic leukaemia	*✓*

*Notes*: Genes with associations to breast cancer are bolded, and genes associated with high-confidence driver mutations are annotated in the rightmost column. Each method was constrained to select exactly 100 genes from a common set. We see that Precision Lasso selects the most relevant genes.

#### 3.2.3 Results

As seen in Table 1, the Precision Lasso effectively selected genes that have been linked to breast cancer. Not only did the Precision Lasso identify at least as many genes with known oncogenic somatic mutations as the baseline methods did, the associations selected by the Precision Lasso are also more likely to be causally related to breast cancer. For example, the Precision Lasso selected *FOXA1* and *AR*, which both have been implicated as potential driver mutations. In contrast, the closest baseline method (Trace Lasso) only selected 1 gene (*FOXA1*) with a somatic mutation known to be associated with breast cancer. While the performance of all methods is likely underestimated due to unknown cancer associations, we expect this effect to be consistent across the sets of variables.

These results suggest that the Precision Lasso can select meaningful variables when the data contains highly correlated or linearly dependent variables, as in the case of breast cancer RNA-seq assays. Furthermore, a gene-specific investigation suggests that while many genes are correlated with breast cancer oncogenesis, few have significant evidence of causality. In this setting, in which there are many variables that are linearly dependent or highly correlated, baseline methods tend to select variables which mediate the causal relationship between genotype and phenotype, as opposed to potential driver mutations that are of more interest to biologists. In contrast, the Precision Lasso tends to select these underlying driver mutations.

## 4 Discussion and Conclusion

While the Precision Lasso has been shown to outperform existing methods on variable selection tasks with highly correlated and linearly dependent data, it is also of interest to compare the relative computational efficiency of the compared methods. Owing to the complexity of the regularizer employed by the Precision Lasso, it is not surprising that it requires more computational resources, scaling cubically with the number of samples and linearly with the number of explanatory variables. Empirically, we find our implementation feasible of a dataset as large as *n *=* *1000 with *p *=* *5000 on a modern laptop (2.60 GHz CPU and 16 G RAM, Linux OS) or *n *=* *5000 with *p *=* *50 000 on a modern server (2.30 GHz CPU and 128 G RAM, Linux OS). Furthermore, even though the Precision Lasso shows improvements in variable selection, it does not necessarily outperform traditional methods such as the Lasso in prediction. This is unsurprising, since the much simpler task of prediction does not suffer from the *unstable* and *inconsistent* problems that are unique to variable selection.

In this paper, we studied the problem of variable selection for genomic data in which a portion of the explanatory variables are either highly correlated or linearly dependent. In this setting, traditional variable selection methods such as the Lasso struggle with *unstable* and *inconsistent* selection. We first showed that these issues are quite real and arise in genomic datasets as a rule rather than an exception. To overcome these challenges, we proposed the Precision Lasso, a novel form of sparse regularization that overcomes many of the drawbacks of traditional methods such as the Lasso. In our experiments, the Precision Lasso outperformed these traditional methods in the presence of highly correlated and linearly dependent variables. With real breast cancer gene expression data, we demonstrated the effectiveness of the Precision Lasso to select more meaningful genes.

The Precision Lasso also offers the potential for extension to other structured methods. In particular, we are interested to see how this variable selection method can help improve structured variable selection methods such as the group lasso (Friedman *et al.*, 2010) and graph fused lasso (Kim and Xing, 2009), as well as population stratification (Wang and Yang, 2016). We are also interested in improving the Precision Lasso when no linearly dependent variables or correlated variables exist. In addition, we are interested to see how Precision Lasso, can help improve predictive performance, akin to Haws *et al.* (2015). In addition, as Bayesian methods are commonly believed to work best when the signal-to-noise ratio (SNR) is small—as is the case in genetic and genomic studies—a Bayesian extension of Precision Lasso is a promising direction for future research.

The Precision Lasso is open-source and freely available as a command line tool that is compatible with either.csv or PLINK files. We also plan to make the methodology available via a point-and-click interface by integrating it in the visual platform GenAMap (Wang *et al.*, 2017).

## Supplementary Material

Supplementary Data
