# Supplementary material for: Precision Lasso: accounting for correlations and linear dependencies in high-dimensional genomic data
Source: Bioinformatics. 2018 Sep 1;35(7):1181–7. doi: 10.1093/bioinformatics/bty750 (PMC6449749; doi:10.1093/bioinformatics/bty750)
Supplement: Supplementary Data [file bty750_supplementary_data.pdf]

Supplementary of *Precision Lasso: Accounting for Correlations and Linear Dependencies in High-Dimensional Genomic Data*

Haohan Wang, Benjamin J. Lengerich, Bryon Aragam, Eric P. Xing

S1 Instructions of Using the Software Precision Lasso

Source code and installation instructions are available at
https://github.com/HaohanWang/thePrecisionLasso.
The software can be used as a stand-alone script without installation, or included in a Python workflow.
Run

```
python runPL.py --help
```

for usage instructions as following:

```
 Options:
-h, --help              Show this help message and exit

 Data Options:
   -t FILETYPE          Input file type, should be one of {plink, }
   -n FILENAME          Name of the input file

 Model Options:
   --lambda=LMBD        The weight of the penalizer. The program will ignore lambda
                        if snum is also set. If neither lambda nor snum is given,
                        cross validation will be run.
   --snum=SNUM          The number of targeted variables the model selects. If
                        neither lambda or snum is given, cross validation will
                        be run.
   -m                   Run without missing genotype imputation
   -b                   Run with logistic regression version

 Advanced Parameter Options:
   --gamma=GAMMA      gamma parameter of the Precision Lasso, if none given, the
                      Precision Lasso will calculate it automatically
   --lr=LR            learning rate of the model
```

Example Usage:

```
python runRL.py -n data/toy -t plink --snum 20
```

This command will use the Precision Lasso to select 20 variables that are associated with the phenotype. Results will be stored in data/toy.output.

GUI implementation A graphical user interface for the Precision Lasso will be available in the software platform GenAMap [1], which is available at http://genamap.org/.

S2 Parameter Learning Algorithm for Precision Lasso

To optimize the parameters of the Precision Lasso, we extend the iteratively re-weighted least-squares method introduced in [2].

To simplify the derivation, we first briefly discuss the iteratively re-weighted least-squares method considering the situation when regularizers are re-weighted by $(X^TX)^{-1}$. The central step of this method is built on the follwoing variational reformulation of the trace norm [3]:

$$\|M\|_\star = \frac{1}{2}\inf_{S\succeq 0} \mathrm{tr}(M^TSM) + \mathrm{tr}(S)$$

and the infimum is attained for $S = (MM^T)^{\frac{1}{2}}$. Using this, we can reformulate the problem in Equation 2 in the main manuscript of the inverse covariance regularizer as:

$$\arg\min_\beta \inf_{S\succeq 0} \frac{1}{2}\|y - X\beta\|_2^2 + \frac{\lambda}{2}\beta^T\mathrm{Diag}(M^TS^{-1}M)\beta + \frac{\lambda}{2}\mathrm{tr}(S) \tag{1}$$

where M is replaced with the actual regularizer:

$$\begin{aligned}\|M\|_\star &= \|(X^TX)^{-\frac{1}{2}}\beta\|_\star \\ &= \|((X\mathrm{diag}(\beta^{-1})^T(X\mathrm{diag}(\beta^{-1}))^{-\frac{1}{2}}\|_\star\end{aligned}$$

and S is solved as following, according to infimum rule of trace norm reformulation.

$$S = (X\mathrm{diag}^2((\beta+\mu)^{-1})X^T + \mu I)^{-\frac{1}{2}} \tag{2}$$

where some small terms μ are added to guarantee a valid inverse.

The problem in Equation 1 is jointly convex in (β, S), so we can proceed to solve for β with S from Equation 2 directly. Solving for β is straightforward. After reformulation as a linear system:

$$(X^TX + \lambda\mathrm{diag}(X^TS^{-1}X))\beta = Xy$$

β can be solved by the traditional solution of linear regression.

However, for Precision Lasso, this solution is not applicable because the linear interpolation of X^TX and $(X^TX)^{-1}$ cannot be easily re-written into a product of two terms. Therefore, we derive the upper bound of the norm and minimize it. The regularizer of Precision Lasso can be upper bounded by the sum of two terms, which corresponds to the regularizer governed by X^TX and the regularizer governed by $(X^TX)^{-1}$ respectively, as shown in the following

$$\begin{aligned}\|M\|_\star =& \|(\gamma(X^TX) + (1-\gamma)(X^TX)^{-1})^{\frac{1}{2}}\beta\|_\star \\ \leq& \|\gamma'(X^TX)^{\frac{1}{2}}\beta \\ &+ (1-\gamma')(X^TX)^{-\frac{1}{2}}\beta\|_\star \\ =& \|\gamma'((X\mathrm{diag}(\beta))^T(X\mathrm{diag}(\beta))^{\frac{1}{2}} \\ &+ (1-\gamma')((X\mathrm{diag}(\beta^{-1})^T(X\mathrm{diag}(\beta^{-1}))^{-\frac{1}{2}}\|_\star \\ =& \gamma'\|((X\mathrm{diag}(\beta))^T(X\mathrm{diag}(\beta))^{\frac{1}{2}}\|_\star \\ &+ (1-\gamma')\|((X\mathrm{diag}(\beta^{-1})^T(X\mathrm{diag}(\beta^{-1}))^{-\frac{1}{2}}\|_\star\end{aligned}$$

Where Line 2 is given from Corollary 2(i) in [4] and Line 4 holds because both X^TX and $(X^TX)^{-1}$ are positive semidefinite and symmetric. γ' is a function of γ to get rid of square root for simplicity.

According to the reformulation of trace norm above, the infimum is attained for

$$\begin{aligned}S =& \gamma(X\mathrm{diag}^2(\beta)X^T)^{\frac{1}{2}} \\ &+ (1-\gamma)(X\mathrm{diag}^2((\beta+\mu)^{-1})X^T + \mu I)^{-\frac{1}{2}}\end{aligned}$$

Similarly to the previous case, in implementation, we add small terms to guarantee there are valid solutions. And we can solve for β with the same solution as in the simple case.

S2.1 Selection of μ

We set the maximum number of iterations of the iterative re-weighted least square algorithm as T. At Iteration $t < T$, we set $\mu^t = 10^{(-2-\frac{8t}{T})}$, which is the same as the original Trace Lasso paper [2]. In practice, we find that the algorithm often converges before T iterations.

S2.2 Extensions of Precision Lasso to other cost functions

The Precision Lasso regularization strategy can be extended to other convex cost functions (e.g. Logistic Regression) in addition to the linear regression cost function investigated above. For example, with a new cost function $\ell(X, y; \beta)$, Equation 1 can be written as:

$$\arg\min_{\beta} \inf_{S \succeq 0} \ell(X, y; \beta) + \frac{\lambda}{2}\beta^T \mathrm{Diag}(M^T S^{-1} M)\beta + \frac{\lambda}{2}\mathrm{tr}(S) \tag{3}$$

As long as this function is jointly convex in (β, S), the iteratively re-weighted least squares method can be applied by solving for β and S alternatively.

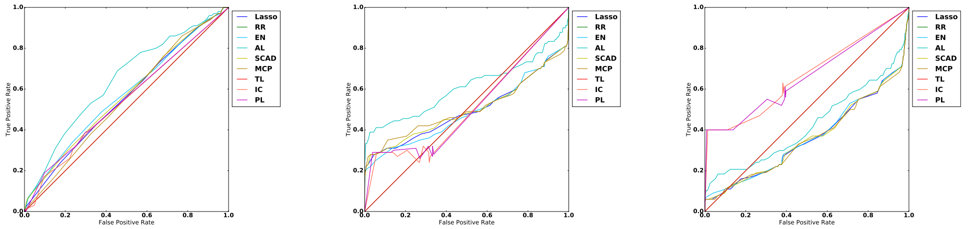

(a) AR=0 (b) AR=0.4 (c) AR=0.8

Figure S1: The ROC curve averaged with 10 random runs for 100 samples and 10 active variables for different autoregressive settings. The figures are shown in the case-control case.

S3 Simulation Data

S3.1 Detailed Data Generation Procedure

To test the performance of Precision Lasso in variable selection that accounts for the correlated and linearly dependent variables, we generate the response matrix $X \in \mathcal{R}^{n\times p}$ with two subsets: correlated variables $X_1 \in \mathcal{R}^{n\times p_1}$, and linearly dependent variables $X_2 \in \mathcal{R}^{n\times p_2}$, with $p = p_1 + p_2$. We use k to denote the number of active variable (variable with non-zero effect size).

Further, X_1 is generated following standard Yule–Walker equations [5]. Specifically, for every active variable t ($t = 1, 2, \dots, k$), we sample $X_{1,t} \in \mathcal{R}^{n,\frac{p_1}{k}}$. For column i, we have:

$$X_{1,t}^{(i)} = \begin{cases} U(0,1), & i = 1 \\ \rho X_{1,t}^{(i-1)} + (1-\rho^2)N(0,1), & i = 2, 3, \dots, \dfrac{p_1}{k} \end{cases}$$

where $\rho = \{0, 0.4, 0.8\}$ is the correlation that we experiment with in our simulations. $X_{1,t}^{(0)}$ is used as active variable.

Then we sample the remaining variables as linearly dependent variables. For $j = 1, 2, \dots, p_2$, we first sample a random integer m, that $1 \le m \le k$. Then we select m out of k active variables (with replacement), say these variables are indexed by q. Then $X_2^{(j)} = \dfrac{1}{m}\sum_q X^{(q)}$.

Now, we sample effect sizes vector β. The non-zero effect sizes are sampled with $U(0, 1)$. Finally, to generate y, we have two cases:

- For continuous responses, we set $y = X\beta + \epsilon$ with $\epsilon \sim N(0, 1)$, so the SNR is $\frac{1}{12}$.
- For binary responses, we first generate $r = X\beta$, and then sample $y = \mathrm{Ber}(g^{-1}(r))$, where $g^{-1}(\cdot)$ is the inverse logit function.

To conduct a comprehensive comparison of the algorithms, we used three different configurations of sample sizes, active variables and autoregressive correlations respectively, resulting in 27 overall configurations. For each configuration, we tested 10 different random seeds, leading to a total of 270 random datasets tested. We fixed the number of total explanatory variables to be $p = 1000$ and sample sizes of $n = 100, 200, 500$ to simulate the high-dimensional regime with $p > n$ frequently found in genomic analysis.

S3.2 ROC Curves

In Figure S1, we plot the ROC curve for different varialbe selection methods with λ ranging from 10^{-10} to 10^{10}. The results indicate the motivation for the Precision Lasso: Traditional methods do well when the correlations between predictors is low, and rapidly deteriorates as the amount of correlation increases. The Precision Lasso, on the other hand, is robust to such increases in the autoregressive correlation. These curves also corroborate our findings on the AUC across different settings (see Sections S3.3 and S3.4).

S3.3 Detailed Evaluation of Binary response (case-control simulation)

Table S1 shows the complete evaluation results of these methods for six different evaluation metrics in addition to what is reported in the main paper: true positive number (TP), false positive number (FP), precision, recall, F1 score and area under ROC curve (AUC). In Table S1, we report each evaluation metric averaged over 10 repeated simulations with different random seeds. We vary the following parameters: auto-correlation coefficient (ρ), number of active variables (k), and number of samples (n). All settings use $p = 1000$ total variables. Note that Ridge Regression (RR) and Trace Lasso (TL) do not have the sparse variable selection property, and that Wald Testing is implemented with standard FDR control. Overall, while the performance of Precision Lasso is inferior to other methods when the autoregressive correlation is low, it is superior in high autoregressive cases.

ρ	k	n	model	TP	FP	precision	recall	F1	AUC
			Wald	0.0	**0.0**	0.5	0.0	0.0	0.5
			SIS	**7.4**	2.6	**0.74**	**0.74**	**0.74**	**0.869**
			Lasso	6.5	3.7	0.671	0.65	0.657	0.824
			EN	6.5	3.7	0.671	0.65	0.657	0.824
			AL	6.3	3.4	0.685	0.63	0.653	0.814
0	10	100	SCAD	6.5	3.2	0.703	0.65	0.673	0.824
			MCP	6.5	3.6	0.671	0.65	0.658	0.824
			IC	2.8	384.4	0.007	0.28	0.014	0.455
			PL	2.8	383.4	0.007	0.28	0.014	0.453
			RR	10.0	990.0	0.01	1.0	0.02	0.844
			TL	10.0	990.0	0.01	1.0	0.02	0.85
			Wald	0.0	**0.0**	0.5	0.0	0.0	0.5
			SIS	**7.3**	2.7	0.73	**0.73**	**0.73**	**0.864**
			Lasso	6.8	2.3	0.754	0.68	0.713	0.839
			EN	6.8	2.3	0.754	0.68	0.713	0.839
			AL	7.0	2.8	0.725	0.7	0.708	0.849
0	10	200	SCAD	7.0	2.3	**0.762**	0.7	0.726	0.849
			MCP	7.0	2.7	0.735	0.7	0.714	0.849
			IC	3.5	323.6	0.011	0.35	0.021	0.522
			PL	3.4	323.2	0.01	0.34	0.02	0.513
			RR	10.0	990.0	0.01	1.0	0.02	0.938
			TL	10.0	990.0	0.01	1.0	0.02	0.94
			Wald	2.0	**0.1**	**0.967**	0.2	0.324	0.6
			SIS	**9.0**	1.0	0.9	**0.9**	**0.9**	**0.95**
			Lasso	8.3	0.3	0.966	0.83	0.891	0.915
			EN	8.3	0.3	0.966	0.83	0.891	0.915
			AL	8.6	0.9	0.914	0.86	0.884	0.93
0	10	500	SCAD	8.6	0.9	0.918	0.86	0.883	0.93
			MCP	8.4	0.6	0.938	0.84	0.883	0.92
			IC	2.9	181.4	0.016	0.29	0.03	0.564
			PL	3.2	185.6	0.017	0.32	0.032	0.572
			RR	10.0	990.0	0.01	1.0	0.02	0.981
			TL	10.0	990.0	0.01	1.0	0.02	0.985
			Wald	0.0	**0.0**	0.5	0.0	0.0	0.5
			SIS	**15.1**	4.9	0.755	**0.755**	**0.755**	**0.877**
			Lasso	14.0	5.3	0.755	0.7	0.723	0.849
			EN	14.0	5.3	0.755	0.7	0.723	0.849
			AL	13.3	4.1	**0.773**	0.665	0.712	0.831
0	20	100	SCAD	13.9	5.2	0.761	0.695	0.722	0.846
			MCP	13.8	5.7	0.73	0.69	0.707	0.844
			IC	6.8	376.3	0.018	0.34	0.034	0.499
			PL	6.9	373.8	0.018	0.345	0.034	0.5
			RR	20.0	980.0	0.02	1.0	0.039	0.887
			TL	20.0	980.0	0.02	1.0	0.039	0.89
			Wald	0.1	**0.0**	0.55	0.005	0.01	0.503
			SIS	**16.6**	3.4	0.83	**0.83**	**0.83**	**0.914**
			Lasso	16.1	3.5	0.824	0.805	0.812	0.902
			EN	16.1	3.6	0.82	0.805	0.81	0.902
			AL	15.5	2.3	**0.88**	0.775	0.822	0.887
0	20	200	SCAD	15.5	4.0	0.811	0.775	0.788	0.887
			MCP	15.7	2.5	0.873	0.785	0.824	0.892
			IC	6.3	320.7	0.019	0.315	0.036	0.519
			PL	6.2	317.9	0.019	0.31	0.035	0.515
			RR	20.0	980.0	0.02	1.0	0.039	0.947
			TL	20.0	980.0	0.02	1.0	0.039	0.953
			Wald	1.2	**0.0**	0.9	0.06	0.11	0.53
			SIS	**17.4**	2.6	0.87	**0.87**	**0.87**	**0.935**
			Lasso	15.7	1.4	0.923	0.785	0.846	0.892
			EN	15.8	1.0	0.943	0.79	0.858	0.895
			AL	15.5	2.0	0.898	0.775	0.829	0.887
0	20	500	SCAD	15.7	1.1	0.937	0.785	0.852	0.892
			MCP	15.7	0.7	**0.958**	0.785	0.861	0.892
			IC	7.5	162.1	0.044	0.375	0.079	0.615
			PL	7.3	162.5	0.043	0.365	0.077	0.608
			RR	20.0	980.0	0.02	1.0	0.039	0.965
			TL	20.0	980.0	0.02	1.0	0.039	0.975
			Wald	0.0	**0.0**	0.5	0.0	0.0	0.5
			SIS	**40.2**	9.8	0.804	**0.804**	0.804	**0.901**
			Lasso	39.5	10.8	0.81	0.79	0.791	0.894
			EN	39.5	10.6	0.813	0.79	0.793	0.894
			AL	37.9	3.4	**0.923**	0.758	**0.831**	0.878
0	50	100							

			SCAD	38.9	5.8	0.879	0.778	0.823	0.888
			MCP	38.8	6.7	0.871	0.776	0.816	0.887
			IC	17.0	335.6	0.048	0.34	0.084	0.517
			PL	16.9	334.5	0.048	0.338	0.084	0.515
			RR	50.0	950.0	0.05	1.0	0.095	0.942
			TL	50.0	950.0	0.05	1.0	0.095	0.942
			Wald	0.0	**0.0**	0.5	0.0	0.0	0.5
			SIS	42.7	7.3	0.854	0.854	0.854	0.926
			Lasso	41.6	3.3	0.935	0.832	0.878	0.915
			EN	41.6	3.3	0.935	0.832	0.878	0.915
			AL	41.7	2.8	**0.941**	0.834	**0.883**	0.916
0	50	200	SCAD	42.1	6.0	0.878	0.842	0.857	0.92
			MCP	**42.9**	11.5	0.793	**0.858**	0.822	**0.928**
			IC	14.0	273.8	0.049	0.28	0.083	0.519
			PL	14.0	273.4	0.049	0.28	0.084	0.517
			RR	50.0	950.0	0.05	1.0	0.095	0.961
			TL	50.0	950.0	0.05	1.0	0.095	0.961
			Wald	0.2	**0.0**	0.6	0.004	0.008	0.502
			SIS	**45.1**	4.9	0.902	**0.902**	0.902	**0.95**
			Lasso	42.4	1.4	0.971	0.848	**0.903**	0.924
			EN	42.4	1.4	0.971	0.848	**0.903**	0.924
			AL	43.6	4.6	0.912	0.872	0.889	0.936
0	50	500	SCAD	41.6	1.8	0.962	0.832	0.891	0.916
			MCP	41.0	1.1	**0.974**	0.82	0.889	0.91
			IC	16.2	128.7	0.112	0.324	0.166	0.605
			PL	16.2	128.2	0.112	0.324	0.167	0.604
			RR	50.0	950.0	0.05	1.0	0.095	0.979
			TL	50.0	950.0	0.05	1.0	0.095	0.978
			Wald	0.0	**0.0**	0.5	0.0	0.0	0.5
			SIS	**6.2**	3.8	**0.62**	**0.62**	**0.62**	**0.809**
			Lasso	5.2	5.1	0.512	0.52	0.514	0.758
			EN	5.2	5.2	0.501	0.52	0.508	0.758
			AL	5.0	4.4	0.535	0.5	0.515	0.748
0.4	10	100	SCAD	5.2	5.3	0.499	0.52	0.507	0.758
			MCP	5.3	5.5	0.489	0.53	0.506	0.763
			IC	4.1	312.0	0.013	0.41	0.025	0.536
			PL	4.2	311.3	0.013	0.42	0.026	0.537
			RR	10.0	990.0	0.01	1.0	0.02	0.853
			TL	10.0	990.0	0.01	1.0	0.02	0.861
			Wald	0.0	**0.0**	0.5	0.0	0.0	0.5
			SIS	**6.1**	3.9	0.61	**0.61**	0.61	**0.804**
			Lasso	5.9	3.8	0.619	0.59	0.6	0.794
			EN	5.9	4.1	0.601	0.59	0.592	0.794
			AL	5.9	3.5	**0.636**	0.59	**0.61**	0.794
0.4	10	200	SCAD	6.0	4.2	0.604	0.6	0.597	0.799
			MCP	5.9	3.9	0.607	0.59	0.595	0.794
			IC	4.3	226.4	0.018	0.43	0.035	0.607
			PL	4.1	226.4	0.018	0.41	0.034	0.593
			RR	10.0	990.0	0.01	1.0	0.02	0.924
			TL	10.0	990.0	0.01	1.0	0.02	0.934
			Wald	2.0	**0.1**	**0.967**	0.2	0.324	0.6
			SIS	7.3	2.7	0.73	0.73	**0.73**	0.864
			Lasso	**7.4**	3.2	0.706	**0.74**	0.719	0.869
			EN	7.3	3.2	0.703	0.73	0.715	0.864
			AL	7.2	2.8	0.723	0.72	0.719	0.859
0.4	10	500	SCAD	7.2	2.6	0.737	0.72	0.727	0.859
			MCP	**7.4**	2.9	0.721	**0.74**	0.728	**0.869**
			IC	3.5	61.1	0.053	0.35	0.092	0.649
			PL	3.7	65.9	0.052	0.37	0.091	0.655
			RR	10.0	990.0	0.01	1.0	0.02	0.954
			TL	10.0	990.0	0.01	1.0	0.02	0.964
			Wald	0.0	**0.0**	0.5	0.0	0.0	0.5
			SIS	**12.0**	8.0	**0.6**	**0.6**	**0.6**	**0.797**
			Lasso	10.2	9.3	0.523	0.51	0.514	0.751
			EN	10.2	9.3	0.523	0.51	0.514	0.751
			AL	10.3	9.2	0.531	0.515	0.52	0.754
0.4	20	100	SCAD	10.4	9.7	0.512	0.52	0.514	0.756
			MCP	10.4	9.7	0.513	0.52	0.514	0.756
			IC	8.0	300.2	0.026	0.4	0.049	0.549
			PL	8.0	301.3	0.026	0.4	0.049	0.546
			RR	20.0	980.0	0.02	1.0	0.039	0.827
			TL	20.0	980.0	0.02	1.0	0.039	0.832
0.4	20	200	Wald	0.1	**0.0**	0.55	0.005	0.01	0.503

			SIS	**13.0**	7.0	0.65	**0.65**	**0.65**	**0.823**
			Lasso	12.1	6.8	0.65	0.605	0.622	0.8
			EN	12.3	7.1	0.641	0.615	0.624	0.805
			AL	11.9	6.2	**0.659**	0.595	0.622	0.795
			SCAD	12.4	7.5	0.633	0.62	0.622	0.808
			MCP	12.0	7.7	0.622	0.6	0.606	0.798
			IC	7.9	223.2	0.034	0.395	0.063	0.592
			PL	7.7	223.2	0.033	0.385	0.061	0.585
			RR	20.0	980.0	0.02	1.0	0.039	0.899
			TL	20.0	980.0	0.02	1.0	0.039	0.911
			Wald	1.2	**0.0**	**0.9**	0.06	0.11	0.53
			SIS	**13.6**	6.4	0.68	**0.68**	**0.68**	**0.838**
			Lasso	12.4	6.6	0.657	0.62	0.635	0.808
			EN	12.5	6.3	0.669	0.625	0.643	0.811
			AL	12.7	5.5	0.703	0.635	0.664	0.816
0.4	20	500	SCAD	13.2	6.3	0.68	0.66	0.666	0.828
			MCP	13.1	6.9	0.661	0.655	0.654	0.825
			IC	8.6	52.6	0.143	0.43	0.214	0.692
			PL	9.0	57.3	0.138	0.45	0.21	0.698
			RR	20.0	980.0	0.02	1.0	0.039	0.937
			TL	20.0	980.0	0.02	1.0	0.039	0.949
			Wald	0.0	**0.0**	0.5	0.0	0.0	0.5
			SIS	**32.8**	17.2	**0.656**	**0.656**	**0.656**	**0.823**
			Lasso	28.2	20.6	0.578	0.564	0.569	0.774
			EN	28.2	20.6	0.578	0.564	0.569	0.774
			AL	27.6	20.5	0.592	0.552	0.568	0.768
0.4	50	100	SCAD	28.9	21.0	0.582	0.578	0.577	0.781
			MCP	29.3	21.8	0.58	0.586	0.579	0.785
			IC	21.1	258.7	0.076	0.422	0.129	0.579
			PL	21.2	258.8	0.076	0.424	0.129	0.578
			RR	50.0	950.0	0.05	1.0	0.095	0.857
			TL	50.0	950.0	0.05	1.0	0.095	0.881
			Wald	0.0	**0.0**	0.5	0.0	0.0	0.5
			SIS	**33.9**	16.1	0.678	**0.678**	0.678	**0.834**
			Lasso	32.2	14.1	0.699	0.644	0.669	0.817
			EN	32.6	14.9	0.688	0.652	0.669	0.821
			AL	32.2	13.3	**0.709**	0.644	0.673	0.818
0.4	50	200	SCAD	33.2	14.3	0.703	0.664	**0.68**	0.827
			MCP	33.8	16.8	0.674	0.676	0.672	0.833
			IC	17.6	182.7	0.088	0.352	0.141	0.592
			PL	17.6	184.4	0.088	0.352	0.14	0.59
			RR	50.0	950.0	0.05	1.0	0.095	0.934
			TL	50.0	950.0	0.05	1.0	0.095	0.934
			Wald	0.2	**0.0**	0.6	0.004	0.008	0.502
			SIS	35.3	14.7	0.706	0.706	**0.706**	0.848
			Lasso	33.4	14.3	0.705	0.668	0.684	0.829
			EN	33.5	13.3	**0.718**	0.67	0.691	0.831
			AL	33.3	15.4	0.689	0.666	0.673	0.828
0.4	50	500	SCAD	36.2	17.2	0.679	0.724	0.699	0.857
			MCP	**36.7**	18.8	0.664	**0.734**	0.696	**0.861**
			IC	19.6	41.0	0.329	0.392	0.354	0.677
			PL	19.5	41.7	0.326	0.39	0.352	0.678
			RR	50.0	950.0	0.05	1.0	0.095	0.949
			TL	50.0	950.0	0.05	1.0	0.095	0.957
			Wald	0.0	**0.0**	**0.5**	0.0	0.0	0.5
			SIS	3.2	6.8	0.32	0.32	**0.32**	**0.657**
			Lasso	2.2	8.1	0.208	0.22	0.213	0.606
			EN	2.2	8.1	0.208	0.22	0.213	0.606
			AL	2.1	7.7	0.213	0.21	0.21	0.601
0.8	10	100	SCAD	2.0	7.8	0.203	0.2	0.201	0.596
			MCP	2.0	7.7	0.213	0.2	0.205	0.596
			IC	**4.3**	124.8	0.033	**0.43**	0.061	0.65
			PL	4.2	121.9	0.033	0.42	0.06	0.645
			RR	10.0	990.0	0.01	1.0	0.02	0.707
			TL	10.0	990.0	0.01	1.0	0.02	0.713
			Wald	0.0	**0.0**	**0.5**	0.0	0.0	0.5
			SIS	3.9	6.1	0.39	0.39	**0.39**	0.692
			Lasso	1.8	7.9	0.183	0.18	0.181	0.586
			EN	1.8	8.0	0.181	0.18	0.18	0.586
			AL	1.8	8.4	0.172	0.18	0.176	0.586
0.8	10	200	SCAD	1.8	7.6	0.19	0.18	0.184	0.586
			MCP	1.8	7.8	0.185	0.18	0.182	0.586
			IC	**4.6**	34.0	0.124	**0.46**	0.193	**0.713**

			PL	4.5	34.6	0.122	0.45	0.189	0.708
			RR	10.0	990.0	0.01	1.0	0.02	0.733
			TL	10.0	990.0	0.01	1.0	0.02	0.742
			Wald	2.0	**0.0**	**1.0**	0.2	0.327	0.6
			SIS	4.0	6.0	0.4	0.4	0.4	0.698
			Lasso	3.4	5.9	0.37	0.34	0.353	0.667
			EN	3.4	5.8	0.376	0.34	0.355	0.667
			AL	3.1	7.3	0.306	0.31	0.307	0.652
0.8	10	500	SCAD	3.5	6.5	0.357	0.35	0.352	0.672
			MCP	3.5	6.9	0.335	0.35	0.341	0.672
			IC	**4.4**	6.1	0.431	**0.44**	0.433	0.718
			PL	**4.4**	5.3	0.471	**0.44**	**0.453**	**0.718**
			RR	10.0	990.0	0.01	1.0	0.02	0.808
			TL	10.0	990.0	0.01	1.0	0.02	0.849
			Wald	0.0	**0.0**	**0.5**	0.0	0.0	0.5
			SIS	5.6	14.4	0.28	0.28	**0.28**	**0.633**
			Lasso	5.3	14.8	0.269	0.265	0.266	0.625
			EN	5.3	14.8	0.269	0.265	0.266	0.625
			AL	5.2	14.1	0.267	0.26	0.263	0.623
0.8	20	100	SCAD	5.3	14.6	0.267	0.265	0.264	0.626
			MCP	5.2	14.6	0.272	0.26	0.265	0.623
			IC	**8.1**	133.8	0.06	0.405	0.104	0.628
			PL	**8.1**	133.3	0.06	**0.405**	0.104	0.627
			RR	20.0	980.0	0.02	1.0	0.039	0.739
			TL	20.0	980.0	0.02	1.0	0.039	0.753
			Wald	0.1	**0.0**	**0.55**	0.005	0.01	0.503
			SIS	7.6	12.4	0.38	0.38	**0.38**	0.684
			Lasso	5.9	12.6	0.314	0.295	0.303	0.642
			EN	5.9	13.2	0.304	0.295	0.298	0.642
			AL	5.7	13.2	0.298	0.285	0.289	0.637
0.8	20	200	SCAD	5.9	14.5	0.286	0.295	0.289	0.641
			MCP	6.0	12.6	0.319	0.3	0.308	0.644
			IC	**8.3**	41.4	0.17	**0.415**	0.24	**0.688**
			PL	8.1	41.6	0.167	0.405	0.235	0.682
			RR	20.0	980.0	0.02	1.0	0.039	0.677
			TL	20.0	980.0	0.02	1.0	0.039	0.712
			Wald	1.2	**0.0**	**0.9**	0.06	0.11	0.53
			SIS	7.9	12.1	0.395	0.395	0.395	0.692
			Lasso	7.0	12.0	0.364	0.35	0.354	0.669
			EN	6.6	11.5	0.362	0.33	0.343	0.66
			AL	7.1	13.2	0.337	0.355	0.345	0.671
0.8	20	500	SCAD	6.9	12.0	0.357	0.345	0.35	0.667
			MCP	6.7	12.6	0.34	0.335	0.336	0.662
			IC	**9.4**	11.7	0.463	**0.47**	**0.464**	**0.73**
			PL	9.1	11.9	0.45	0.455	0.449	0.723
			RR	20.0	980.0	0.02	1.0	0.039	0.785
			TL	20.0	980.0	0.02	1.0	0.039	0.816
			Wald	0.0	**0.0**	**0.5**	0.0	0.0	0.5
			SIS	16.0	34.0	0.32	0.32	**0.32**	**0.644**
			Lasso	14.3	35.1	0.303	0.286	0.293	0.626
			EN	14.3	34.6	0.306	0.286	0.295	0.626
			AL	14.2	34.2	0.301	0.284	0.292	0.625
0.8	50	100	SCAD	14.6	36.1	0.299	0.292	0.294	0.628
			MCP	14.0	34.6	0.306	0.28	0.29	0.623
			IC	**22.9**	136.4	0.146	**0.458**	0.221	0.643
			PL	**22.9**	137.0	0.145	**0.458**	0.22	0.639
			RR	50.0	950.0	0.05	1.0	0.095	0.641
			TL	50.0	950.0	0.05	1.0	0.095	0.648
			Wald	0.0	**0.0**	**0.5**	0.0	0.0	0.5
			SIS	19.4	30.6	0.388	0.388	**0.388**	0.68
			Lasso	16.6	29.1	0.366	0.332	0.347	0.653
			EN	16.1	28.0	0.367	0.322	0.342	0.648
			AL	17.5	29.5	0.377	0.35	0.361	0.661
0.8	50	200	SCAD	17.8	31.6	0.361	0.356	0.357	0.663
			MCP	17.1	29.3	0.368	0.342	0.354	0.657
			IC	**20.8**	48.9	0.301	**0.416**	0.348	**0.683**
			PL	20.7	52.1	0.288	0.414	0.338	0.679
			RR	50.0	950.0	0.05	1.0	0.095	0.815
			TL	50.0	950.0	0.05	1.0	0.095	0.814
			Wald	0.2	**0.0**	**0.6**	0.004	0.008	0.502
			SIS	20.4	29.6	0.408	0.408	0.408	0.691
			Lasso	19.2	26.6	0.42	0.384	0.4	0.68
			EN	19.2	26.9	0.417	0.384	0.399	0.68
0.8	50	500							

AL	20.7	31.5	0.399	0.414	0.405	0.693
SCAD	20.0	29.7	0.408	0.4	0.402	0.687
MCP	19.9	27.7	0.419	0.398	0.406	0.687
IC	**25.4**	20.9	0.554	**0.508**	**0.528**	**0.744**
PL	24.6	27.6	0.483	0.492	0.485	0.733
RR	50.0	950.0	0.05	1.0	0.095	0.842
TL	50.0	950.0	0.05	1.0	0.095	0.845

Table S1: Complete comparison of methods on simulated case-control data. Notice that RR and TL are evaluated separately because they selected every variable.

S3.4 Detailed Evaluation of Continuous Response

In addition to the simulation of case-control study, we also generate the simulation data where the response variable y are continuous variables. The data is generated following the protocol described in Section S3.1. As Figure S2 and Table S2 show, the results of the continuous case are favors more of Precision Lasso, especially in high correlation case. Please notice that the comparison excludes RR and TL because these two methods do not produce sparse solution.

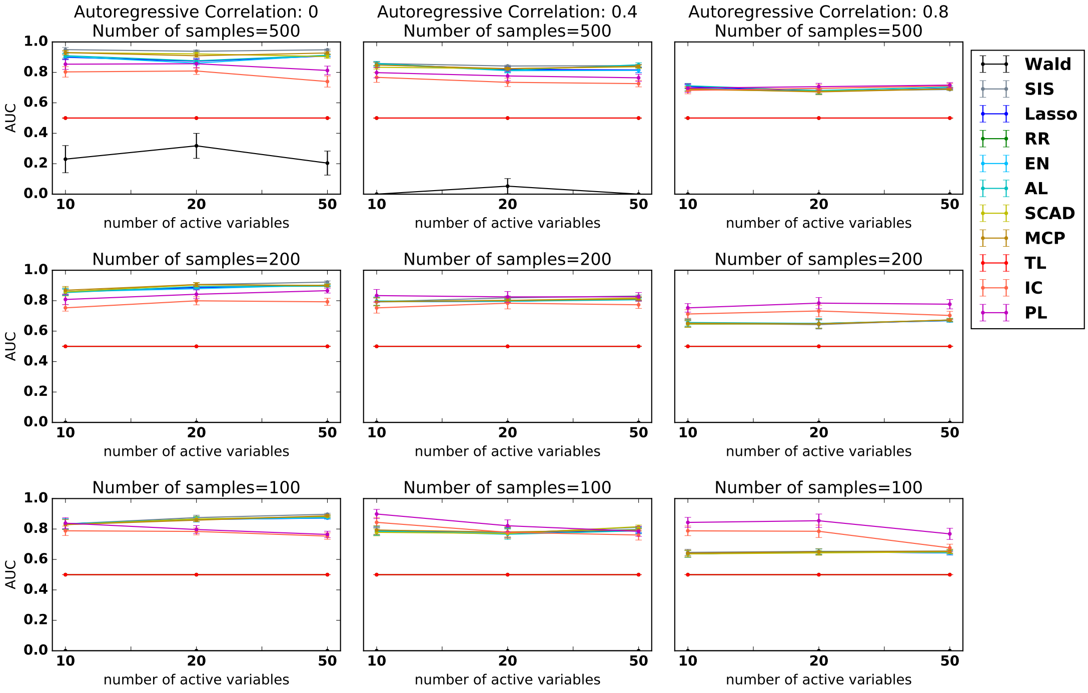

Figure S2: AUC of each variable selection method.

ρ	k	n	model	TP	FP	precision	recall	F1	AUC
			Wald	0.0	**0.0**	0.5	0.0	0.0	0.5
			SIS	6.7	3.3	0.67	0.67	0.67	0.834
			Lasso	6.7	3.2	0.688	0.67	0.675	0.834
			EN	6.7	3.3	0.68	0.67	0.672	0.834
			AL	6.7	2.7	0.725	0.67	0.694	0.834
0	10	100	SCAD	6.6	2.2	**0.763**	0.66	**0.706**	0.829
			MCP	6.6	2.8	0.722	0.66	0.686	0.829
			IC	5.8	3.7	0.627	0.58	0.601	0.788
			PL	**6.8**	3.4	0.678	**0.68**	0.678	**0.839**
			RR	10.0	990.0	0.01	1.0	0.02	0.876
			TL	10.0	990.0	0.01	1.0	0.02	0.929
			Wald	0.0	**0.0**	0.5	0.0	0.0	0.5
			SIS	7.2	2.8	0.72	0.72	0.72	0.859
			Lasso	7.2	3.4	0.69	0.72	0.701	0.859
			EN	7.2	3.1	0.709	0.72	0.71	0.859
			AL	7.1	3.4	0.689	0.71	0.695	0.854
0	10	200	SCAD	7.2	2.8	0.726	0.72	0.72	0.859
			MCP	**7.4**	2.4	**0.771**	**0.74**	**0.751**	**0.869**
			IC	5.1	3.8	0.573	0.51	0.538	0.754
			PL	6.2	3.9	0.605	0.62	0.611	0.809
			RR	10.0	990.0	0.01	1.0	0.02	0.949
			TL	10.0	990.0	0.01	1.0	0.02	0.934
			Wald	0.6	**0.0**	0.7	0.06	0.103	0.53
			SIS	**9.0**	1.0	0.9	**0.9**	**0.9**	**0.95**
			Lasso	8.0	0.8	0.925	0.8	0.856	0.9
			EN	8.1	1.2	0.892	0.81	0.845	0.905
			AL	8.2	0.7	0.926	0.82	0.866	0.91
0	10	500	SCAD	8.6	0.5	**0.949**	0.86	0.899	0.93
			MCP	8.6	0.7	0.936	0.86	0.892	0.93
			IC	6.1	3.5	0.622	0.61	0.613	0.804
			PL	7.1	2.2	0.779	0.71	0.74	0.854
			RR	10.0	990.0	0.01	1.0	0.02	0.98
			TL	10.0	990.0	0.01	1.0	0.02	0.93
			Wald	0.0	**0.0**	0.5	0.0	0.0	0.5
			SIS	**15.1**	4.9	0.755	**0.755**	0.755	**0.877**
			Lasso	14.6	4.6	0.776	0.73	0.747	0.864
			EN	14.6	4.9	0.765	0.73	0.742	0.864
			AL	14.7	3.6	0.817	0.735	0.769	0.867
0	20	100	SCAD	14.7	3.3	**0.827**	0.735	**0.775**	0.867
			MCP	14.4	4.1	0.802	0.72	0.754	0.859
			IC	11.5	6.9	0.637	0.575	0.602	0.784
			PL	12.0	6.6	0.655	0.6	0.624	0.797
			RR	20.0	980.0	0.02	1.0	0.039	0.902
			TL	20.0	980.0	0.02	1.0	0.039	0.95
			Wald	0.0	**0.0**	0.5	0.0	0.0	0.5
			SIS	**16.3**	3.7	0.815	**0.815**	0.815	0.907
			Lasso	15.5	2.0	0.897	0.775	0.828	0.887
			EN	15.2	2.3	0.885	0.76	0.813	0.88
			AL	15.8	3.8	0.826	0.79	0.803	0.894
0	20	200	SCAD	16.2	2.0	**0.9**	0.81	**0.848**	0.905
			MCP	**16.3**	2.7	0.866	**0.815**	0.836	**0.907**
			IC	12.1	6.5	0.671	0.605	0.631	0.8
			PL	13.8	5.5	0.725	0.69	0.703	0.843
			RR	20.0	980.0	0.02	1.0	0.039	0.954
			TL	20.0	980.0	0.02	1.0	0.039	0.929
			Wald	0.7	**0.0**	0.8	0.035	0.066	0.518
			SIS	**17.6**	2.4	0.88	**0.88**	**0.88**	**0.94**
			Lasso	15.0	2.5	0.882	0.75	0.806	0.874
			EN	14.5	2.7	0.87	0.725	0.786	0.862
			AL	15.1	2.2	0.891	0.755	0.815	0.877
0	20	500	SCAD	16.9	2.0	0.906	0.845	0.869	0.922
			MCP	16.4	1.5	**0.931**	0.82	0.866	0.91
			IC	12.5	6.5	0.681	0.625	0.649	0.811
			PL	14.4	6.2	0.703	0.72	0.71	0.859
			RR	20.0	980.0	0.02	1.0	0.039	0.965
			TL	20.0	980.0	0.02	1.0	0.039	0.929
			Wald	0.0	**0.0**	0.5	0.0	0.0	0.5
			SIS	**40.2**	9.8	0.804	**0.804**	0.804	**0.901**
			Lasso	37.4	3.9	**0.913**	0.748	0.821	0.873
			EN	37.5	4.1	0.91	0.75	0.821	0.874
			AL	38.5	9.2	0.818	0.77	0.79	0.884
0	50	100							

			SCAD	38.5	4.7	0.899	0.77	**0.827**	0.884
			MCP	39.1	7.5	0.849	0.782	0.81	0.89
			IC	26.5	22.0	0.546	0.53	0.535	0.753
			PL	27.6	22.5	0.554	0.552	0.549	0.764
			RR	50.0	950.0	0.05	1.0	0.095	0.938
			TL	50.0	950.0	0.05	1.0	0.095	0.945
			Wald	0.0	**0.0**	0.5	0.0	0.0	0.5
			SIS	**42.6**	7.4	0.852	**0.852**	0.852	**0.925**
			Lasso	40.4	3.9	0.916	0.808	0.857	0.903
			EN	40.6	5.1	0.894	0.812	0.849	0.905
			AL	39.7	3.2	0.931	0.794	0.855	0.896
0	50	200	SCAD	40.2	2.1	**0.952**	0.804	**0.87**	0.902
			MCP	40.0	2.1	0.952	0.8	0.868	0.9
			IC	30.3	19.6	0.613	0.606	0.606	0.795
			PL	37.2	11.7	0.768	0.744	0.753	0.867
			RR	50.0	950.0	0.05	1.0	0.095	0.958
			TL	50.0	950.0	0.05	1.0	0.095	0.911
			Wald	0.4	**0.0**	0.7	0.008	0.016	0.504
			SIS	**45.1**	4.9	0.902	**0.902**	**0.902**	**0.95**
			Lasso	40.8	1.1	0.975	0.816	0.888	0.908
			EN	41.2	1.0	**0.977**	0.824	0.893	0.912
			AL	41.4	2.5	0.947	0.828	0.882	0.914
0	50	500	SCAD	40.5	1.9	0.958	0.81	0.875	0.905
			MCP	42.9	2.8	0.947	0.858	0.897	0.929
			IC	25.4	26.7	0.505	0.508	0.504	0.744
			PL	32.0	13.6	0.72	0.64	0.676	0.815
			RR	50.0	950.0	0.05	1.0	0.095	0.975
			TL	50.0	950.0	0.05	1.0	0.095	0.913
			Wald	0.0	**0.0**	0.5	0.0	0.0	0.5
			SIS	5.9	4.1	0.59	0.59	0.59	0.794
			Lasso	5.7	4.3	0.582	0.57	0.573	0.784
			EN	5.7	4.3	0.582	0.57	0.573	0.784
			AL	5.8	4.7	0.558	0.58	0.567	0.789
0.4	10	100	SCAD	5.6	3.9	0.607	0.56	0.58	0.779
			MCP	5.9	4.0	0.603	0.59	0.594	0.794
			IC	6.9	2.6	0.729	0.69	0.704	0.844
			PL	**8.0**	2.9	**0.737**	**0.8**	**0.767**	**0.899**
			RR	10.0	990.0	0.01	1.0	0.02	0.89
			TL	10.0	990.0	0.01	1.0	0.02	0.983
			Wald	0.0	**0.0**	0.5	0.0	0.0	0.5
			SIS	5.9	4.1	0.59	0.59	0.59	0.794
			Lasso	5.9	4.3	0.589	0.59	0.587	0.794
			EN	5.9	4.4	0.582	0.59	0.583	0.794
			AL	6.0	4.1	0.605	0.6	0.599	0.799
0.4	10	200	SCAD	5.9	3.5	0.626	0.59	0.604	0.794
			MCP	5.9	3.6	0.626	0.59	0.604	0.794
			IC	5.1	4.4	0.556	0.51	0.53	0.753
			PL	**6.7**	3.0	**0.687**	**0.67**	**0.675**	**0.834**
			RR	10.0	990.0	0.01	1.0	0.02	0.933
			TL	10.0	990.0	0.01	1.0	0.02	0.938
			Wald	0.0	**0.0**	0.5	0.0	0.0	0.5
			SIS	**7.2**	2.8	0.72	**0.72**	**0.72**	0.859
			Lasso	7.1	2.8	**0.724**	0.71	0.714	0.854
			EN	7.1	3.0	0.712	0.71	0.708	0.854
			AL	**7.2**	3.3	0.692	**0.72**	0.703	**0.859**
0.4	10	500	SCAD	6.7	2.9	0.708	0.67	0.685	0.834
			MCP	7.0	3.0	0.703	0.7	0.698	0.849
			IC	5.4	5.2	0.51	0.54	0.524	0.769
			PL	6.0	3.3	0.645	0.6	0.619	0.799
			RR	10.0	990.0	0.01	1.0	0.02	0.963
			TL	10.0	990.0	0.01	1.0	0.02	0.935
			Wald	0.0	**0.0**	0.5	0.0	0.0	0.5
			SIS	11.1	8.9	0.555	0.555	0.555	0.774
			Lasso	11.2	9.6	0.531	0.56	0.544	0.777
			EN	11.2	9.6	0.531	0.56	0.544	0.777
			AL	10.8	8.3	0.569	0.54	0.552	0.767
0.4	20	100	SCAD	11.0	8.5	0.563	0.55	0.554	0.772
			MCP	11.4	8.9	0.563	0.57	0.566	0.782
			IC	11.3	8.3	0.596	0.565	0.578	0.78
			PL	**13.0**	7.0	**0.662**	**0.65**	**0.653**	**0.823**
			RR	20.0	980.0	0.02	1.0	0.039	0.857
			TL	20.0	980.0	0.02	1.0	0.039	0.903
			Wald	0.0	**0.0**	0.5	0.0	0.0	0.5

0.4 20 200

			SIS	12.9	7.1	0.645	0.645	0.645	0.82
			Lasso	12.0	6.8	0.655	0.6	0.621	0.798
			EN	12.0	6.8	0.655	0.6	0.621	0.798
			AL	12.0	7.4	0.632	0.6	0.612	0.798
			SCAD	12.2	6.3	0.666	0.61	0.633	0.803
			MCP	12.2	6.4	0.663	0.61	0.632	0.803
			IC	11.5	7.9	0.6	0.575	0.584	0.785
			PL	**13.1**	5.0	**0.722**	**0.655**	**0.685**	**0.826**
			RR	20.0	980.0	0.02	1.0	0.039	0.897
			TL	20.0	980.0	0.02	1.0	0.039	0.941
			Wald	0.1	**0.0**	0.55	0.005	0.01	0.503
			SIS	**13.8**	6.2	0.69	**0.69**	**0.69**	**0.843**
			Lasso	12.9	7.4	0.64	0.645	0.639	0.82
			EN	12.6	7.1	0.646	0.63	0.634	0.813
			AL	12.7	7.2	0.649	0.635	0.638	0.815
0.4	20	500	SCAD	13.1	5.6	**0.718**	0.655	0.679	0.826
			MCP	13.1	5.9	0.696	0.655	0.672	0.826
			IC	9.6	10.3	0.496	0.48	0.485	0.736
			PL	11.2	6.9	0.647	0.56	0.599	0.778
			RR	20.0	980.0	0.02	1.0	0.039	0.941
			TL	20.0	980.0	0.02	1.0	0.039	0.883
			Wald	0.0	**0.0**	0.5	0.0	0.0	0.5
			SIS	32.1	17.9	0.642	0.642	0.642	0.815
			Lasso	30.2	20.0	0.612	0.604	0.607	0.795
			EN	30.8	20.3	0.612	0.616	0.613	0.801
			AL	29.9	20.6	0.596	0.598	0.594	0.792
0.4	50	100	SCAD	**32.5**	18.4	0.643	**0.65**	**0.644**	**0.818**
			MCP	30.2	14.5	**0.681**	0.604	0.638	0.797
			IC	27.1	19.8	0.589	0.542	0.563	0.763
			PL	29.6	21.3	0.602	0.592	0.594	0.787
			RR	50.0	950.0	0.05	1.0	0.095	0.884
			TL	50.0	950.0	0.05	1.0	0.095	0.919
			Wald	0.0	**0.0**	0.5	0.0	0.0	0.5
			SIS	**33.9**	16.1	0.678	**0.678**	0.678	**0.834**
			Lasso	31.9	14.2	0.7	0.638	0.665	0.815
			EN	32.0	14.5	0.695	0.64	0.664	0.815
			AL	31.3	12.9	**0.712**	0.626	0.663	0.809
0.4	50	200	SCAD	32.5	13.9	0.7	0.65	0.673	0.821
			MCP	31.9	14.5	0.695	0.638	0.662	0.815
			IC	28.5	22.6	0.56	0.57	0.564	0.777
			PL	33.3	13.9	0.707	0.666	**0.683**	0.829
			RR	50.0	950.0	0.05	1.0	0.095	0.93
			TL	50.0	950.0	0.05	1.0	0.095	0.943
			Wald	0.0	**0.0**	0.5	0.0	0.0	0.5
			SIS	35.2	14.8	**0.704**	0.704	**0.704**	0.847
			Lasso	32.4	14.9	0.691	0.648	0.666	0.82
			EN	32.1	14.7	0.692	0.642	0.664	0.817
			AL	**35.9**	17.5	0.679	**0.718**	0.694	**0.854**
0.4	50	500	SCAD	34.5	15.4	0.696	0.69	0.69	0.841
			MCP	34.8	15.3	0.699	0.696	0.696	0.844
			IC	23.9	24.5	0.504	0.478	0.488	0.729
			PL	27.6	22.1	0.576	0.552	0.561	0.768
			RR	50.0	950.0	0.05	1.0	0.095	0.952
			TL	50.0	950.0	0.05	1.0	0.095	0.892
			Wald	0.0	**0.0**	0.5	0.0	0.0	0.5
			SIS	3.0	7.0	0.3	0.3	0.3	0.647
			Lasso	2.8	7.3	0.275	0.28	0.275	0.637
			EN	2.8	7.3	0.275	0.28	0.275	0.637
			AL	2.8	7.4	0.267	0.28	0.272	0.637
0.8	10	100	SCAD	2.8	7.1	0.277	0.28	0.277	0.637
			MCP	2.9	7.4	0.283	0.29	0.285	0.642
			IC	5.8	4.1	0.604	0.58	0.589	0.789
			PL	**6.9**	3.4	**0.685**	**0.69**	**0.684**	**0.844**
			RR	10.0	990.0	0.01	1.0	0.02	0.808
			TL	10.0	990.0	0.01	1.0	0.02	0.952
			Wald	0.0	**0.0**	0.5	0.0	0.0	0.5
			SIS	3.1	6.9	0.31	0.31	0.31	0.652
			Lasso	3.0	6.5	0.338	0.3	0.317	0.647
			EN	3.0	6.7	0.335	0.3	0.315	0.647
			AL	3.2	6.8	0.323	0.32	0.32	0.657
0.8	10	200	SCAD	3.0	7.0	0.315	0.3	0.306	0.647
			MCP	3.1	6.9	0.322	0.31	0.315	0.652
			IC	4.3	4.7	0.474	0.43	0.449	0.713

			PL	**5.1**	4.3	**0.555**	**0.51**	**0.53**	**0.754**
			RR	10.0	990.0	0.01	1.0	0.02	0.828
			TL	10.0	990.0	0.01	1.0	0.02	0.897
			Wald	0.0	**0.0**	**0.5**	0.0	0.0	0.5
			SIS	3.9	6.1	0.39	0.39	0.39	0.692
			Lasso	4.2	6.4	0.399	0.42	0.408	0.707
			EN	4.0	6.0	0.401	0.4	0.399	0.698
			AL	**4.3**	6.0	0.424	**0.43**	**0.425**	**0.712**
0.8	10	500	SCAD	3.8	6.2	0.383	0.38	0.38	0.688
			MCP	3.9	6.1	0.393	0.39	0.39	0.693
			IC	3.7	6.4	0.372	0.37	0.37	0.682
			PL	4.0	6.4	0.4	0.4	0.398	0.698
			RR	10.0	990.0	0.01	1.0	0.02	0.896
			TL	10.0	990.0	0.01	1.0	0.02	0.873
			Wald	0.0	**0.0**	0.5	0.0	0.0	0.5
			SIS	6.4	13.6	0.32	0.32	0.32	0.654
			Lasso	6.3	14.0	0.303	0.315	0.307	0.651
			EN	6.3	14.0	0.303	0.315	0.307	0.651
			AL	6.3	13.1	0.335	0.315	0.322	0.651
0.8	20	100	SCAD	6.0	13.7	0.313	0.3	0.305	0.644
			MCP	6.3	14.2	0.298	0.315	0.304	0.651
			IC	11.6	9.7	0.543	0.58	0.559	0.787
			PL	**14.3**	6.3	**0.699**	**0.715**	**0.702**	**0.856**
			RR	20.0	980.0	0.02	1.0	0.039	0.797
			TL	20.0	980.0	0.02	1.0	0.039	0.925
			Wald	0.0	**0.0**	0.5	0.0	0.0	0.5
			SIS	6.0	14.0	0.3	0.3	0.3	0.644
			Lasso	6.3	14.5	0.297	0.315	0.304	0.651
			EN	6.3	14.7	0.297	0.315	0.304	0.651
			AL	6.3	13.2	0.319	0.315	0.315	0.652
0.8	20	200	SCAD	6.2	13.7	0.317	0.31	0.313	0.649
			MCP	6.2	14.0	0.305	0.31	0.306	0.649
			IC	9.5	10.0	0.513	0.475	0.491	0.734
			PL	**11.5**	6.9	**0.652**	**0.575**	**0.609**	**0.785**
			RR	20.0	980.0	0.02	1.0	0.039	0.753
			TL	20.0	980.0	0.02	1.0	0.039	0.912
			Wald	0.0	**0.0**	**0.5**	0.0	0.0	0.5
			SIS	7.4	12.6	0.37	0.37	0.37	0.679
			Lasso	7.2	12.9	0.365	0.36	0.361	0.674
			EN	7.2	12.5	0.365	0.36	0.361	0.675
			AL	7.5	12.7	0.364	0.375	0.367	0.682
0.8	20	500	SCAD	7.3	13.9	0.356	0.365	0.358	0.677
			MCP	7.1	11.3	0.386	0.355	0.368	0.673
			IC	8.0	10.3	0.436	0.4	0.416	0.696
			PL	**8.5**	12.4	0.421	**0.425**	**0.419**	**0.708**
			RR	20.0	980.0	0.02	1.0	0.039	0.863
			TL	20.0	980.0	0.02	1.0	0.039	0.843
			Wald	0.0	**0.0**	0.5	0.0	0.0	0.5
			SIS	17.2	32.8	0.344	0.344	0.344	0.657
			Lasso	15.9	30.5	0.345	0.318	0.33	0.645
			EN	16.0	30.9	0.343	0.32	0.33	0.646
			AL	16.9	32.1	0.352	0.338	0.343	0.654
0.8	50	100	SCAD	16.7	33.2	0.34	0.334	0.335	0.652
			MCP	17.0	30.2	0.363	0.34	0.35	0.656
			IC	19.3	32.1	0.37	0.386	0.377	0.68
			PL	**27.8**	18.9	**0.597**	**0.556**	**0.575**	**0.771**
			RR	50.0	950.0	0.05	1.0	0.095	0.774
			TL	50.0	950.0	0.05	1.0	0.095	0.923
			Wald	0.0	**0.0**	0.5	0.0	0.0	0.5
			SIS	19.0	31.0	0.38	0.38	0.38	0.676
			Lasso	18.4	29.4	0.388	0.368	0.377	0.67
			EN	18.6	31.4	0.373	0.372	0.372	0.672
			AL	18.7	29.1	0.392	0.374	0.381	0.674
0.8	50	200	SCAD	18.9	30.2	0.385	0.378	0.381	0.675
			MCP	18.8	29.8	0.388	0.376	0.38	0.674
			IC	21.9	30.3	0.415	0.438	0.425	0.707
			PL	**28.6**	17.1	**0.626**	**0.572**	**0.596**	**0.78**
			RR	50.0	950.0	0.05	1.0	0.095	0.83
			TL	50.0	950.0	0.05	1.0	0.095	0.902
			Wald	0.0	**0.0**	0.5	0.0	0.0	0.5
			SIS	20.3	29.7	0.406	0.406	0.406	0.69
			Lasso	21.2	31.3	0.406	0.424	0.414	0.699
			EN	20.9	29.2	0.42	0.418	0.417	0.696
0.8	50	500							

AL	21.8	27.0	0.452	0.436	0.441	0.706
SCAD	20.7	28.4	0.425	0.414	0.417	0.696
MCP	20.7	29.9	0.413	0.414	0.412	0.695
IC	22.5	25.6	0.484	0.45	0.464	0.714
PL	**22.8**	22.1	**0.518**	**0.456**	**0.483**	**0.719**
RR	50.0	950.0	0.05	1.0	0.095	0.863
TL	50.0	950.0	0.05	1.0	0.095	0.88

Table S2: Complete comparison of methods with simulated continuous response. Notice that RR and TL are evaluated separately because they selected every variable.

S4 Combined Simulation and Real Data

S4.1 Data Generation

To create the combined data, we use real gene expression data, methylation data and miRNA data for three different cancers, resulting in 9 data sets in total, all from the TCGA Research Network.[1] The goal of this data set is to evaluate performance via F1 scores while retaining distributions of real genomic data. To accomplish this goal, we randomly select 1% of the variables to be active variables and assign random effect sizes to these variables from a normal distribution.

S4.2 Results

Following the same strategy of the Experiment 1 in the main paper, we selected the parameters λ and γ to select the model that identifies a fixed number of associated variables. Similarly, γ is chosen as the proportion of correlated variables over linear dependent variables. Again, we run the experiment ten times and report the averaged F1 score with the standard error.

As Figure S3 shows, Precision Lasso outperforms other methods across these data sets, except two cases: Breast cancer with methylation data and Lung cancer with methylation data. Interestingly, these two exceptional cases are the only cases where traditional hypothesis testing via the Wald Test is optimal. In addition, we observe that all the traditional variable selection methods tend to have larger variance for these two data sets. These observations indicate the difficulty of variable selection for these data sets, and suggests an explanation for the performance decrease of the Precision Lasso in these cases.

S5 Experiments for Breast Cancer Gene Expression Data

To set a ground truth for the variable selection task, we used the COSMIC catalogue of somatic mutations in cancer [6], which lists known oncogenes and their associated tumor types. Overall, this set contained 602 genes, of which 296 were included in the set of 10000 genes being tested. Hyperparameter optimization was performed independently for each model by matching the number of selected genes to equal 100. When methods were not able to select exactly 100 genes, we selected the 100 genes with the maximum absolute value of effect sizes.

[1] http://cancergenome.nih.gov/

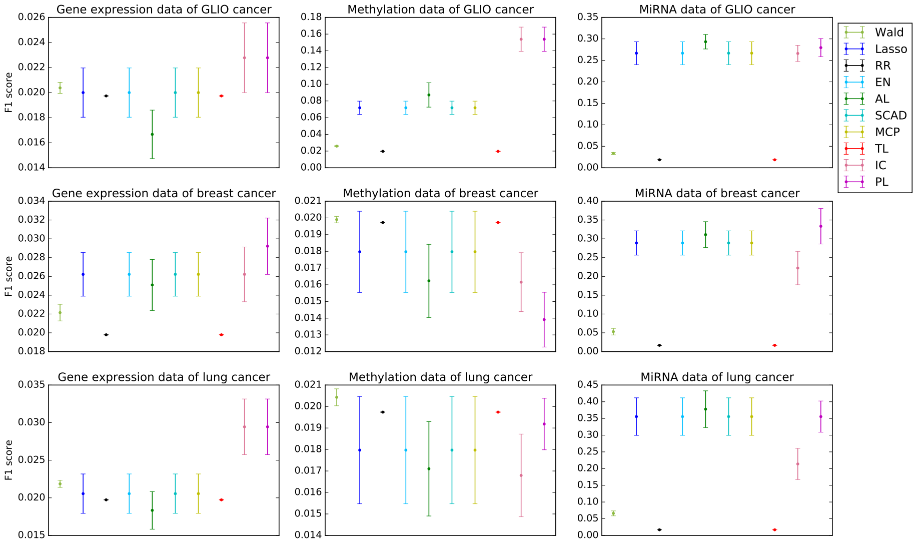

Figure S3: F1 score of each variable selection method. Methods are: Lasso, Ridge Regression (RR), Elastic Net (EN), Adaptive Lasso (AL), SCAD, MCP, Trace Lasso (TL), Inverse Covariance Regularizer (IC) and Precision Lasso (PL). The vertical axis stands for F1 score of the variable selection. The results are averaged from ten runs and standard deviation is also shown. From the plot we can see that our proposed methods (PL and IC) have a clear advantage over traditional methods in most cases.

S6 Comparison of Different Parameter Tuning Strategies

Finally, we compare the different parameter tuning method we showed previous for simulation data. The scores are calculated for each method and each model as an average over all random seeds across all 27 experimental settings. Table S3 shows the result. Interestingly, these results reflect that cross-validation tends to select a lot more variables than our approach. While cross-validation methods can report more true positives, the resulting false positive rates of these methods should be noticed.

References

[1] H. Wang, B. J. Lengerich, M. K. Lee, and E. P. Xing, "Genamap on web: Visual machine learning for next-generation genome wide association studies," *submitted*, 2017.

[2] E. Grave, G. R. Obozinski, and F. R. Bach, "Trace lasso: a trace norm regularization for correlated designs," in *Advances in Neural Information Processing Systems*, pp. 2187–2195, 2011.

[3] A. Evgeniou and M. Pontil, "Multi-task feature learning," *Advances in neural information processing systems*, vol. 19, p. 41, 2007.

[4] T. Ando, "Comparison of norms $|||f(a) - f(b)|||$ and $|||f(|a-b|)|||$," *Mathematische Zeitschrift*, vol. 197, no. 3, pp. 403–409, 1988.

[5] S. Theodoridis, *Machine learning: a Bayesian and optimization perspective.* Academic Press, 2015.

[6] S. A. Forbes, D. Beare, P. Gunasekaran, K. Leung, N. Bindal, H. Boutselakis, M. Ding, S. Bamford, C. Cole, S. Ward, C. Y. Kok, M. Jia, T. De, J. W. Teague, M. R. Stratton, U. McDermott, and P. J. Campbell, "Cosmic: exploring the world's knowledge of somatic mutations in human cancer," *Nucleic Acids Research*, vol. 43, no. D1, pp. D805–D811, 2015.

Table S3: Comparison of different parameter, including selecting a fixed number of variables (when variables are mis-specified) and cross validation (5 fold and 10 fold) tuning strategy for these methods. Numbers are reported across all experimental settings. These numbers are reported in the case-control experiment setting.

Tuning Strategy	Model	TP	FP	precision	recall	F1	AUC
Select $k/2$ Variables	Lasso	4.81	6.40	0.46	0.17	0.24	0.58
	RR	26.67	973.33	0.03	1.00	0.05	0.41
	EN	6.50	5.74	0.54	0.24	0.33	0.62
	AL	3.75	7.51	0.32	0.11	0.15	0.55
	SCAD	5.49	7.29	0.40	0.19	0.25	0.59
	MCP	5.34	7.31	0.39	0.18	0.24	0.58
	TL	26.67	973.33	0.03	1.00	0.05	0.57
	IC	10.19	58.64	0.40	0.37	0.32	0.66
	PL	10.19	69.31	0.36	0.37	0.30	0.65
Select k Variables	Lasso	15.32	9.92	0.59	0.56	0.57	0.77
	RR	26.67	973.33	0.03	1.00	0.05	0.86
	EN	15.31	9.87	0.59	0.56	0.57	0.77
	AL	15.29	9.86	0.60	0.55	0.57	0.77
	SCAD	15.57	10.33	0.59	0.56	0.57	0.78
	MCP	15.55	10.48	0.59	0.56	0.57	0.78
	TL	26.67	973.33	0.03	1.00	0.05	0.87
	IC	10.37	174.17	0.13	0.39	0.16	0.61
	PL	10.31	174.74	0.12	0.39	0.15	0.61
Select $2k$ Variables	Lasso	6.05	38.36	0.21	0.20	0.15	0.58
	RR	26.67	973.33	0.03	1.00	0.05	0.42
	EN	7.76	38.93	0.20	0.28	0.21	0.62
	AL	6.61	36.74	0.20	0.18	0.14	0.58
	SCAD	8.38	43.77	0.13	0.26	0.17	0.61
	MCP	8.26	44.57	0.13	0.25	0.16	0.60
	TL	26.67	973.33	0.03	1.00	0.05	0.57
	IC	9.83	77.46	0.14	0.35	0.19	0.64
	PL	9.91	87.03	0.14	0.35	0.19	0.64
5 fold CV	Lasso	18.73	612.63	0.12	0.60	0.05	0.50
	RR	26.67	973.33	0.03	1.00	0.05	0.57
	EN	18.22	602.55	0.11	0.58	0.04	0.51
	AL	18.23	611.56	0.07	0.59	0.04	0.50
	SCAD	19.13	634.36	0.20	0.63	0.05	0.51
	MCP	18.43	637.13	0.19	0.61	0.05	0.50
	TL	26.67	973.33	0.03	1.00	0.05	0.48
	IC	17.00	715.43	0.10	0.64	0.11	0.52
	PL	17.03	722.01	0.10	0.64	0.11	0.51
10 fold CV	Lasso	18.80	619.67	0.12	0.60	0.05	0.51
	RR	26.67	973.33	0.03	1.00	0.05	0.53
	EN	18.30	609.53	0.11	0.58	0.04	0.51
	AL	18.30	619.08	0.07	0.60	0.04	0.50
	SCAD	19.19	640.19	0.20	0.64	0.05	0.51
	MCP	18.50	644.36	0.19	0.61	0.05	0.50
	TL	26.67	973.33	0.03	1.00	0.05	0.48
	IC	17.03	712.90	0.10	0.64	0.11	0.52
	PL	17.16	717.95	0.10	0.65	0.11	0.52
